# Brazilian Consortium for the Study on Renal Diseases Associated With COVID-19: A Multicentric Effort to Understand SARS-CoV-2-Related Nephropathy

**DOI:** 10.3389/fmed.2020.584235

**Published:** 2020-10-23

**Authors:** Antonio Augusto Lima Teixeira Júnior, Precil Diego Miranda de Menezes Neves, Joyce Santos Lages, Kaile de Araújo Cunha, Monique Pereira Rêgo Muniz, Dyego José de Araújo Brito, Andréia Watanabe, Elieser Hitoshi Watanabe, Luiz Fernando Onuchic, Lucas Lobato Acatauassu Nunes, Antônio Fernando Coutinho Filho, Flávia Lara Barcelos, Giuseppe Cesare Gatto, Antonio Monteiro, Diego do Amaral Polido, Douglas Rafanelle Moura de Santana Motta, Thaísa de Oliveira Leite, Felipe Leite Guedes, Orlando Vieira Gomes, Lucila Maria Valente, Karla Cristina Silva Petruccelli Israel, Francisco Rasiah Ladchumananandasivam, Lígia Cristina Lopes de Farias, Igor Denizarde Bacelar Marques, Gustavo Lemos Uliano, Carlos Eduardo Campos Maramaldo, Lídio Gonçalves Lima Neto, Weverton Machado Luchi, David Campos Wanderley, Stanley de Almeida Araújo, Natalino Salgado Filho, Gyl Eanes Barros Silva

**Affiliations:** ^1^Postgraduate Program in Genetics, Ribeirão Preto Medical School, University of São Paulo (PGGEN-FMRP-USP), Ribeirão Preto, Brazil; ^2^University Hospital, Federal University of Maranhão (HU-UFMA), São Luís, Brazil; ^3^Clinical Research Center (CEPEC), University Hospital, Federal University of Maranhão (HU-UFMA), São Luís, Brazil; ^4^Nephrology Division, Medical School, University of São Paulo (FM-USP), São Paulo, Brazil; ^5^Molecular Medicine Discipline, Medical School, University of São Paulo (FM-USP), São Paulo, Brazil; ^6^Nephrology and Dialysis Center, Oswaldo Cruz German Hospital, São Paulo, Brazil; ^7^Nephrology Service, University Hospital, Federal University of Maranhão (HU-UFMA), São Luís, Brazil; ^8^University Hospital Complex, João de Barros Barreto University Hospital, Federal University of Pará (HUJBB – UFPA), Belém, Brazil; ^9^Dr. Raimundo Bezerra Unit of Dialysis and Kidney Transplantation (UNIRIM), Crato, Brazil; ^10^Nephrology Service, University Hospital of Brasilia, University of Brasília (HUB – UNB), Brasília, Brazil; ^11^Maria Aparecida Pedrossian University Hospital, Federal University of Mato Grosso do Sul (HUMAP – UFMS), Campo Grande, Brazil; ^12^University Hospital, Federal University of Grande Dourados (HU-UFGD), Dourados, Brazil; ^13^Nephrology Service, University Hospital of the Federal University of Sergipe (HU-UFS), Aracaju, Brazil; ^14^Nephrology Service, University Hospital of Lizard, Federal University of Sergipe (HUL - UFS), Lagarto, Brazil; ^15^Nephrology Service, Onofre Lopes University Hospital, Federal University of Rio Grande do Norte (HUOL-UFRN), Natal, Brazil; ^16^University Hospital, Federal University of Vale do São Francisco (HU-UNIVASF), Petrolina, Brazil; ^17^Faculty of Medicine, Federal University of Pernambuco (UFPE), Recife, Brazil; ^18^Postgraduate Program in Tropical Medicine (FMT/UEA), Department of Clinical Medicine, Federal University of Amazonas (UFAM), Manaus, Brazil; ^19^Nephrology Service, Lauro Wanderley University Hospital, Federal University of Paraíba (HULW-UFPB), João Pessoa, Brazil; ^20^Medical Clinic Unit, Hospital Universitário Alcides Carneiro (HUAC), Campina Grande, Brazil; ^21^Nephrology Service, University Hospital, Federal University of Piauí (HU-UFPI), Teresina, Brazil; ^22^School-Hospital, Federal University of Pelotas (HE-UFPEL), Pelotas, Brazil; ^23^Laboratory of Immunology and Microbiology of Respiratory Infections (LIMIR), Maranhão University Center (CEUMA), São Luís, Brazil; ^24^Nephrology Service, Cassiano Antonio Moraes University Hospital, Federal University of Espírito Santo (HUCAM-UFES), Vitória, Brazil; ^25^Nephropathology Institute, Federal University of Minas Gerais (UFMG), Belo Horizonte, Brazil

**Keywords:** COVID-19, SARS-CoV-2, glomerulopathy, kidney injury, collapsing glomerulopathy, thrombotic microangiopathy

## Abstract

Kidney involvement appears to be frequent in coronavirus disease 2019 (COVID-19). Despite this, information concerning renal involvement in COVID-19 is still scarce. Several mechanisms appear to be involved in the complex relationship between the virus and the kidney. Also, different morphological patterns have been described in the kidneys of patients with COVID-19. For some authors, however, this association may be just a coincidence. To investigate this issue, we propose assessing renal morphology associated with COVID-19 at the renal pathology reference center of federal university hospitals in Brazil. Data will come from a consortium involving 17 federal university hospitals belonging to *Empresa Brasileira de Serviços Hospitalares* (EBSERH) network, as well as some state hospitals and an autopsy center. All biopsies will be sent to the referral center for renal pathology of the EBSERH network. The data will include patients who had coronavirus disease, both alive and deceased, with or without pre-existing kidney disease. Kidney biopsies will be analyzed by light, fluorescence, and electron microscopy. Furthermore, immunohistochemical (IHC) staining for various inflammatory cells (i.e., cells expressing CD3, CD20, CD4, CD8, CD138, CD68, and CD57) as well as angiotensin-converting enzyme 2 (ACE2) will be performed on paraffinized tissue sections. In addition to ultrastructural assays, *in situ* hybridization (ISH), IHC and reverse transcription-polymerase chain reaction (RT-PCR) will be used to detect Severe Acute Respiratory Syndrome Coronavirus (SARS-CoV-2) in renal tissue. For the patients diagnosed with Collapsing Glomerulopathy, peripheral blood will be collected for apolipoprotein L-1 (APOL1) genotyping. For patients with thrombotic microangiopathy, thrombospondin type 1 motif, member 13 (ADAMTS13), antiphospholipid, and complement panel will be performed. The setting of this study is Brazil, which is second behind the United States in highest confirmed cases and deaths. With this complete approach, we hope to help define the spectrum and impact, whether immediate or long-term, of kidney injury caused by SARS-CoV-2.

## Background

The coronavirus disease 2019 (COVID-19) caused by the severe acute respiratory syndrome coronavirus 2 (SARS-CoV-2) has rapidly spread globally. Currently, Brazil has the second highest number of deaths in the world, and is considered an epicenter of the disease (1). The respiratory system is the primary target of the virus, but it is increasingly recognized to manifest in other organs, including the kidneys. In fact, kidney involvement is relatively common with this infection: 59% of patients present with proteinuria, 44% with hematuria, and 10–14% with azotemia. This is associated with increased morbidity and mortality, as this acute renal injury can be fatal, especially when associated with other underlying diseases (2).

Numerous morphological patterns have been described in the kidneys of patients with COVID-19, including renal infarction (3–5), collapsing glomerulopathy (CG) (6–10), tubulointerstitial nephritis (11), acute tubular injury, (2, 12, 13) acute pyelonephritis, rhabdomyolysis, lymphocytic infiltrates, and focal segmental glomerulosclerosis (14), isometric vacuoles in the proximal tubular epithelium (12, 14) and thrombotic microangiopathy (TMA) (15).

Despite this, information concerning renal involvement in COVID-19 is still scarce. Several mechanisms appear to be involved in a complex process driven by many factors, including the virus directly causing injury, cytokine storms, angiotensin-II pathway activation, complement disorders, hypercoagulation states, and microangiopathy interacting known risk factors for acute kidney injury (AKI) (16). A more recent report on 17 patients, most with glomerular disease, favored cytokine-mediated effects and adaptive immune responses, rather than direct viral action, as the main mechanism underlying the lesions associated with SARs-CoV-2. With respect to acute renal injury, the mechanism appears to have multiple origins (17). However, there are still effects of SARS-CoV-2 on the kidneys that occur due currently unknown mechanisms. Therefore, any morphological changes should be described using imaging assays (e.g., light, fluorescent, and electron microscopy).

Furthermore, in patients with COVID-19, the morphological patterns of kidney damage may be influenced by the ethnic composition of each country (10). It is worth noting that almost 40% of the Brazilian population has African ancestry, which makes Brazil the country with the second-highest percentage of people of African descent worldwide (18). African-American individuals have a 3- to 4-fold higher risk of end-stage renal disease (ESRD) as compared to white individuals (19). This may be due to the prevalence of a high-risk genotype of apolipoprotein L-1 gene (*APOL1*) in this ethnic group. Collapsing glomerulopathy is a severe form of kidney disease related to *APOL1* and has been described as COVID-19–associated glomerulopathy (6–10).

To address all of these issues, a consortium will be conducted involving 17 federal university hospitals belonging to the *Empresa Brasileira de Serviços Hospitalares* (EBSERH) network. In addition, some other state hospitals and an autopsy center in São Luís (MA, Brazil), will be participating. The aim of this project is to assess renal morphology associated with COVID-19 at the renal pathology reference center of federal university hospitals in Brazil.

## Methods

### Research Sites and Analysis

The EBSERH network was created to manage 42 federal hospitals located in all states of Brazil. One of its objectives is to create reference centers for specific services to support other hospitals. In many of the Brazilian states, university hospitals are the only places that perform renal biopsies for state's public network. Because of its adequate structure and technical qualification, the Laboratory of Immunofluorescence and Electron Microscopy (LIME) at University Hospital of the Federal University of Maranhão (HUUFMA), located in São Luís (MA, Brazil), was chosen as a reference center for renal pathology. Therefore, the biopsies that are collected in all hospital units will be sent to LIME-HUUFMA for the analysis needed for this study. They will all be evaluated by 03 independently nephropathologist (GS, SA, and DW).

**Figure 1:**
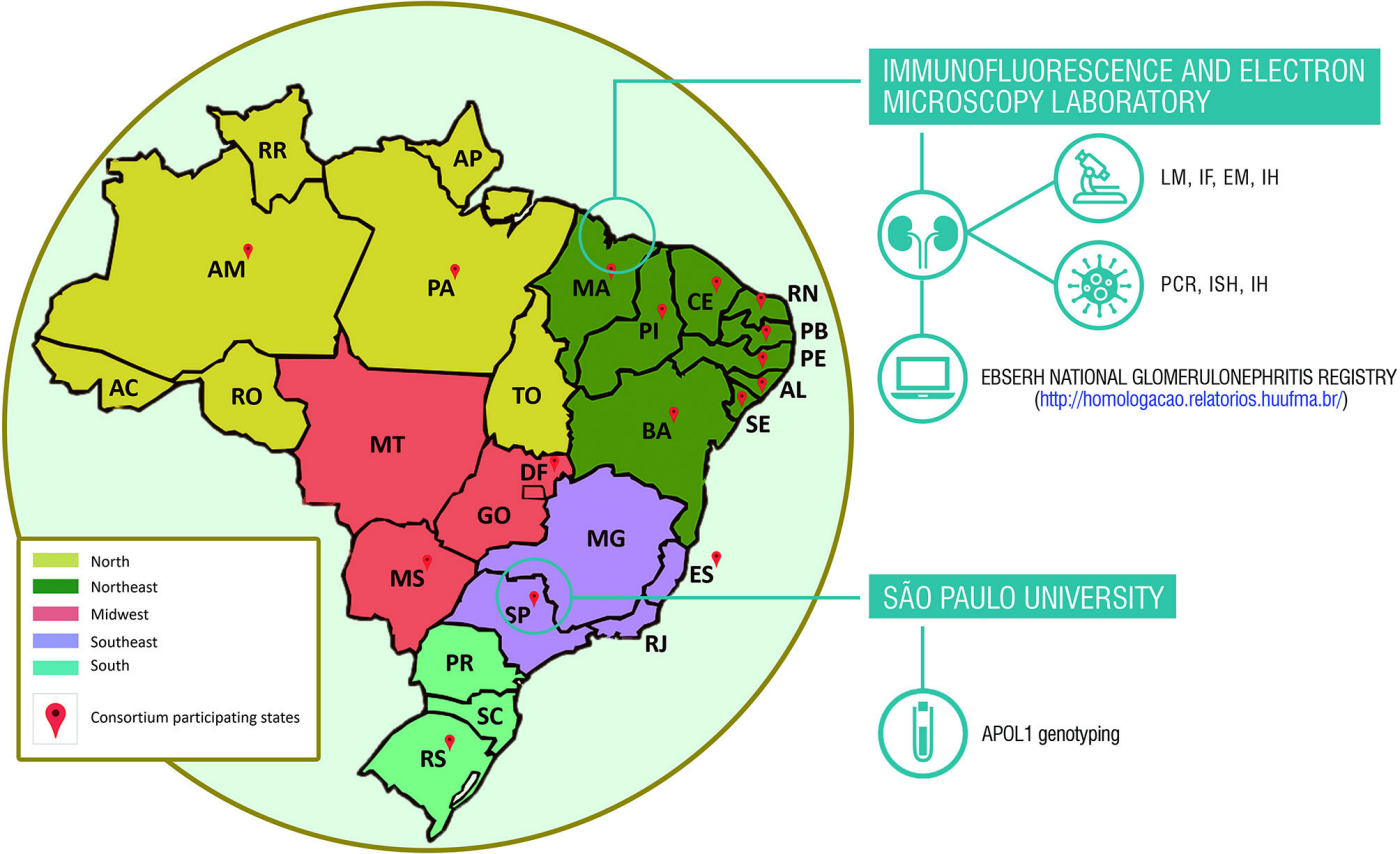
Brazilian states consortium for studies of renal diseases associated with coronavirus disease (COVID-19). APOL1, apolipoprotein L1 gene; EM, electron microscopy; IF, immunofluorescence; IH, immunohistochemistry; ISH, *in situ* hybridization; LM, light microscopy; PCR, polymerase chain reaction.

The study will be conducted in collaboration with 24 Brazilian health service providers that are distributed around country, representing 14 states of the federation plus the federal district. There will be 17 federal university hospitals, 6 state hospitals and one center of postmortem evaluation contributing to the study (Figure 1).

All findings will be report in a largely descriptive fashion.

### Deceased Patients

**Figure 2:**
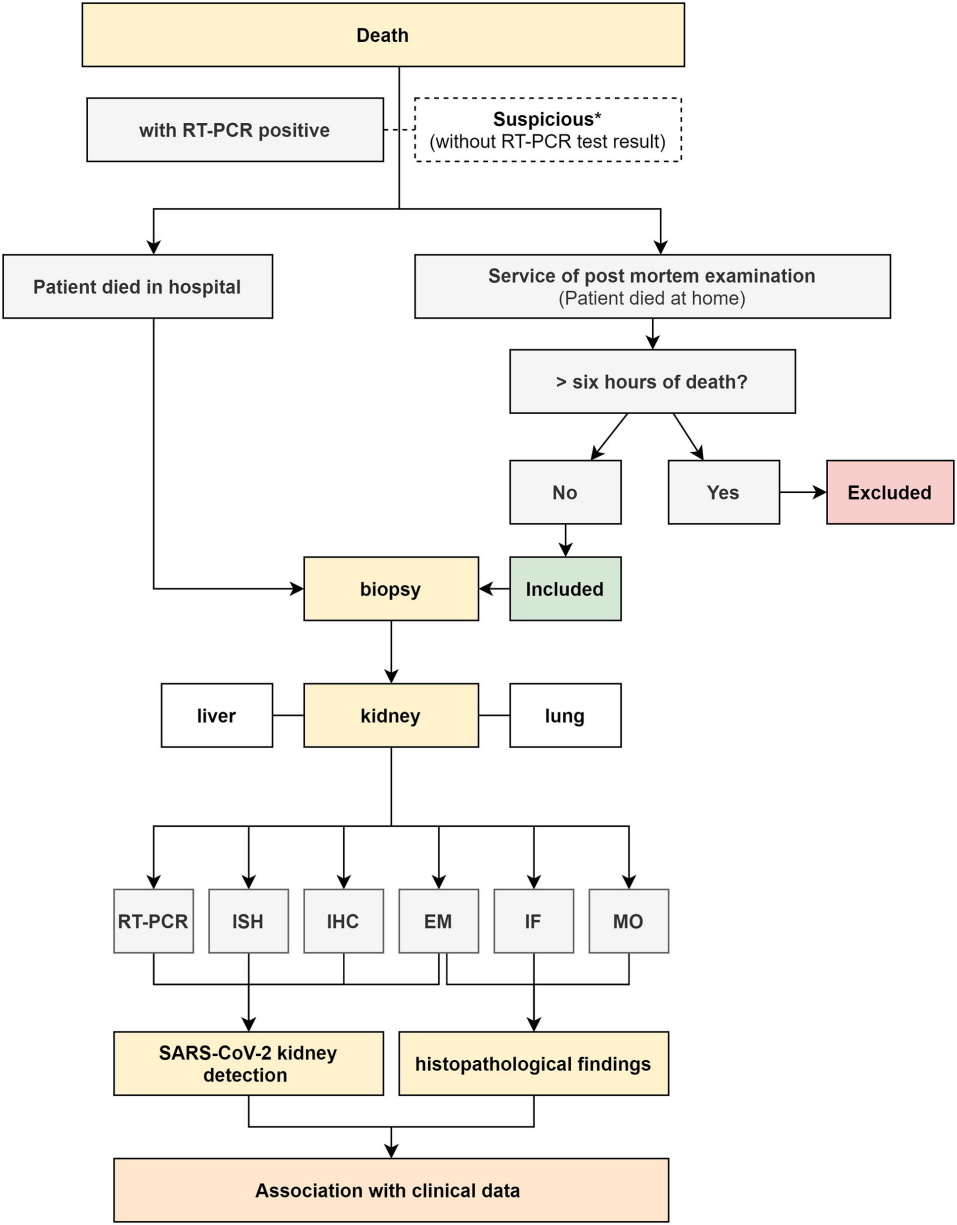
Flowchart of collection and processing of death biopsies associated with COVID-19. RT-PCR (Reverse-Transcriptase Polymerase Chain Reaction); ISH (*in situ* hybridization); IHC (immunohistochemistry); MO (light microscopy); IF (immunofluorescence); EM (electron microscopy); *(collected and discarded due to a negative RT-PCR result).

Cases of deceased patients will come from five public hospitals in Maranhão, Brazil: Federal Hospital of the Federal University of Maranhão (HUUFMA), Women's Hospital, Integrated Clinics Hospital, Real Hospital, and Dr. Carlos Macieira Hospital. For patients who died at home, a center that performs postmortem evaluation (the Maranhão Death Verification Service, SVO-MA) will also be involved. In these cases, lung and liver biopsies will be collected for comparison with the changes found in the kidneys. Placental tissue will be collected from stillborn infants. A consent form signed by the appropriate family member was a mandatory inclusion criterion for the study. For assessing the renal tissue of the patients who had died, individuals of any age with a positive PCR test result for COVID-19 were included, regardless of the presence or absence of dysfunction. Patients whose tissue samples were collected owing to clinical suspicion of COVID-19-related death, but produced negative PCR test results subsequently were excluded. Figure 2 illustrates the work flowchart for cases of deceased patients.

### Living Patients

**Figure 3:**
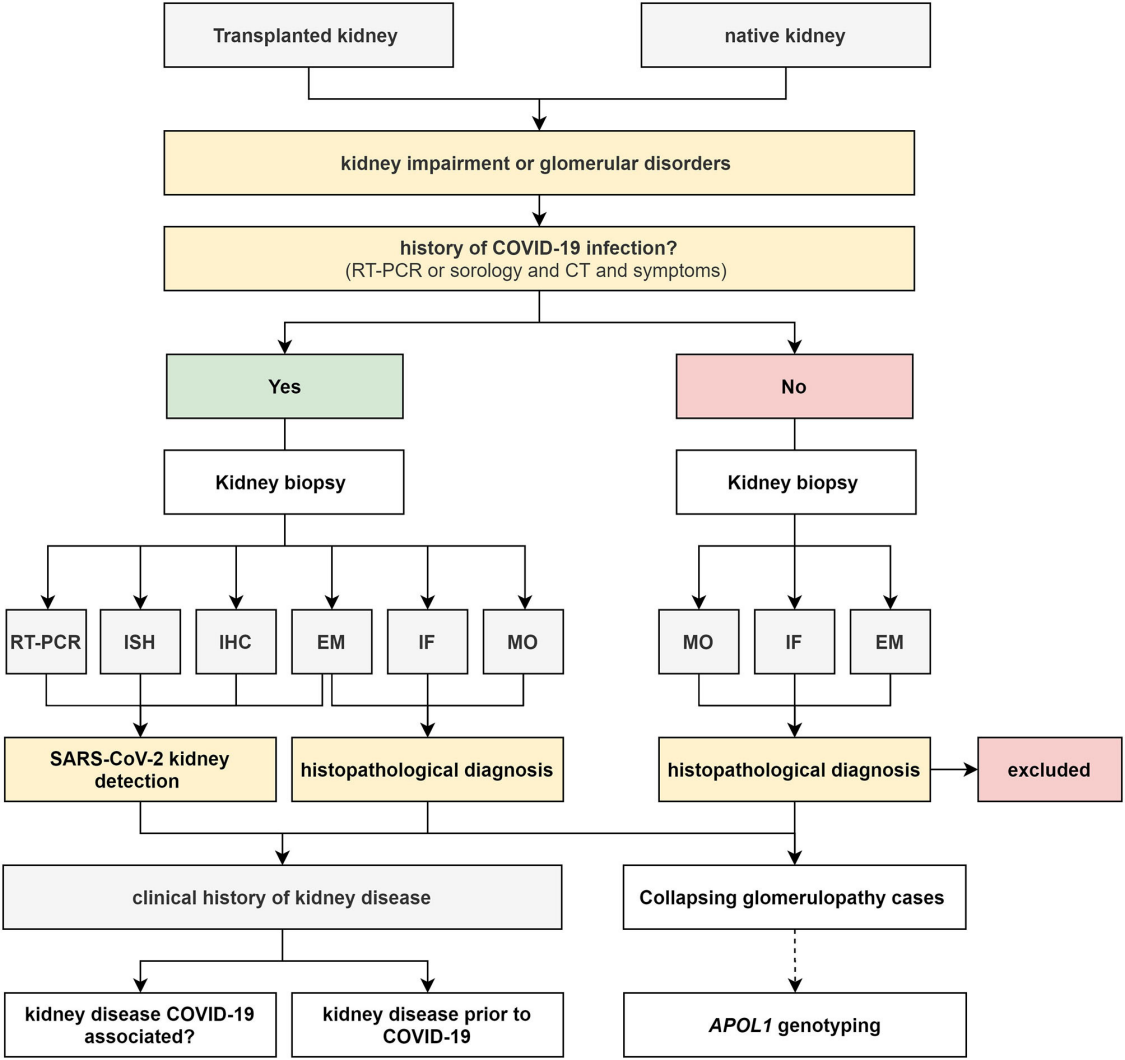
Flowchart of the collection and processing of living patients. RT-PCR (Reverse-Transcriptase Polymerase Chain Reaction); ISH (*in situ* hybridization); IHC (immunohistochemistry); MO (light microscopy); IF (immunofluorescence); EM (electron microscopy).

Cases of living patients will come from 17 federal hospitals of EBSERH, representing all five regions of the country. The diagnosis of COVID-19 will be confirmed by performing RT-PCR on samples (nasopharyngeal secretion or bronchoalveolar lavage fluids), detecting the presence of SARS-CoV-2–specific antibodies, examining radiological features, and clinical symptoms. To standardize renal biopsy requests, an electronic histopathological examination request system for renal biopsies was created in 2019 to act as a national renal biopsy registry (http://homologacao.relatorios.huufma.br/). In this system, most information is mandatory, such as age, race, hometown, personal and family history, clinical history, laboratory data (urinalysis, erythrocyte dysmorphism, blood count, urea, creatinine, 24-h proteinuria, serology, autoantibodies, and fractions complement), medications, imaging tests performed and their results, and clinical syndrome with diagnostic hypothesis. For this project, some mandatory requirements regarding COVID-19 were also added: the patient must have had some infectious symptom during the epidemic, results from serological tests, RT-PCR for SARS-CoV-2, and imaging exams. Information on whether kidney symptoms came before or after COVID-19 disease should also be provided. Therefore, patients infected with SARS-CoV-2 will be divided into two groups: those who did not have any kidney symptoms before contracting COVID-19 and those who already had symptoms compatible with pre-existing kidney disease. In the second group, it is important to evaluate the impact of the disease on kidneys, which produce different pathologies. All of these same assessments will be performed on patients with kidney transplant. For the profile of kidney disease established by biopsies from all of these groups will be compared with data from the pre-epidemic periods of COVID-19. For the patients diagnosed with CG, peripheral blood will be collected for apolipoprotein L-1 (APOL1) genotyping. For patients with TAM, thrombospondin type 1 motif, member 13 (ADAMTS13), antiphospholipid and complement panel will be performed. Other possible causes of TMA, such as autoimmune diseases, CMV infections, EBV, and arboviruses, among others, will be excluded by using specific tests. The workflow chart for living patients is described in Figure 3 and summarized in Figure 1.

After signing the consent form, individuals of any age with a positive PCR test result for SARs-CoV-2 with an indication for renal biopsy were included in the study. Patients with clinical suspicion of COVID-19 with a negative PCR test result were excluded. Patients who presented with renal symptoms before, during, or after SARs-CoV-2 infection were included in the study for various assessments. Patients who had a significant time interval between infection and renal biopsy were accepted if they had the appropriate clinical history, requisite tomography findings, and positive serology for SARs-CoV-2. Patients who did not meet all three criteria were excluded.

#### Biopsy Technique, Tissue Sampling, and Processing

Ultrasound-guided percutaneous renal biopsy using 14-gauge end-cut (for deceased patients) or 16-gauge needles (for living patients) will be performed by nephrologists. In living patients, three samples will be taken. In deceased patients, four samples will be taken from each kidney with a postmortem interval ranging from 1 to 6 h. Tissue specimens will be fixed in Bouin solution for light microscopy (MO), Michel solution for immunofluorescence (IF), and modified formalin or 2.5% glutaraldehyde for electron microscopy (EM). RNAlater solution will be used for tissue preservation for RT-PCR reaction for deceased patients. Only samples with 10 or more glomeruli and at least two arteries were included in the study. Staining for IgG, IgA, IgM, C3, C1q, fibrinogen, kappa, and lambda will be performed using IF techniques on frozen samples from kidney biopsies. Hematoxylin and eosin, periodic acid–Schiff, Masson trichrome, and Jones methenamine silver staining will be performed on paraffin-imbedded sections for MO analysis.

#### Detection of SARS-CoV-2 in the Kidney

To detect SARS-CoV-2 in renal tissue, we will use immunohistochemistry (IHC) to detect antigens and *in situ* hybridization (ISH) and reverse-transcriptase polymerase chain reaction (RT-PCR) to detect the virus's genetic material. Samples that produce a positive result in any of the assays will be considered positive. In addition, these findings will be compared with data obtained from electron microscopy, which will also be used for virus detection.

### Immunohistochemistry (IHC)

For IHC, a Dako Envision™ kit and PT Link (DAKO) equipment will be used for all assays. Three micrometer-thick histological sections will be made from biopsies samples and placed on slides suitable for IHC. The slides will be incubated in an oven for 30 min at 60°C to remove excess paraffin. The slides will then be immersed in a recovery solution containing Dako Target Retrieval Solution (10 ×) diluted 1:10 in distilled water and temperature cycled in the PT Link equipment according to the protocol provided by the supplier (i.e., pre-heating to 65°C; antigen recovery at 97°C for 20 min; cooling to 65°C). The slides will subsequently be washed in Dako Wash Buffer (1 ×) for 5 min. After washing, the following steps are performed: endogenous peroxidase block (5 min), application of monoclonal primary antibody against SARS-CoV-2 (1:1,000, 40143-T62; Sino Biological, Beijing, China), polymer application (20 min), 3,3′ Diaminobenzidine (DAB) chromogen staining (10 min), Harris Hematoxylin counter-staining (5 min), and slide assembly for optical microscope analysis. Staining for various inflammatory cells (i.e., cells that are positive for CD3, CD20, CD4, CD8, CD138, CD68, and/or CD57) and angiotensin-converting enzyme 2 (ACE2) (Abcam, ab15348; Cambridge, MA, USA) will be also performed. For diagnostic purposes, some samples will be stained with antibodies against C4d, amyloid A, CD61, phospholipase A2 receptor (PLA2R), CD44, Wilms's tumor 1 (WT1), etc.

### *In situ* Hybridization (ISH)

For detection of SARS-CoV-2 RNA in kidney tissue by ISH, the RNAscope™ technology (ACD, Newark, CA, USA) as previously described by Wang et al. (20). Larsen et al. (10) used this RNAscope to demonstrate that a COVID-19 positive patient with collapsing glomerulopathy did not show presence of the SARS-CoV-2 virus in the kidney biopsy.

### RT-PCR

For detection of SARS-CoV-2 in the kidney, RNA extraction will be performed from the biopsy samples preserved in RNAlater solution (Invitrogen, ThermoFisher Scientific, Waltham, MA, USA). The AllPrep DNA/RNA/miRNA Universal kit (Qiagen, Hilden, Germany) will be used for RNA isolation. RNA concentration will be measured using the NanoDrop ND-1000 spectrophotometer (NanoDrop Technologies, Rockland, DE, USA), at wavelengths of 230, 260, and 280 nm. The RNA integrity will be then evaluated on a 1.5% agarose gel. cDNA synthesis will be performed using the High Capacity cDNA Reverse Transcription Kit (Applied Biosystems, Foster City, CA, USA). The samples will subsequently be subjected to detection of SARS-CoV-2 by RT-PCR, using the protocol CDC 2019-Novel Coronavirus (2019-nCoV) Real-Time RT-PCR Diagnostic Panel. This protocol uses oligonucleotide primers and probes that were selected from regions of the nucleocapsid (N) virus gene. The panel was designed for specific detection of 2019-nCoV and includes two primer/probe sets. An additional primer/probe set will be used to detect the human RNase P gene (*RP*). All tests will be performed according to the manufacturer's specifications.

### *APOL1* Genotyping of Collapsing Glomerulopathy (CG) Cases

Blood samples will be collected after detailed explanation of the project and its implications to the patient or relatives and after informed consent is obtained. The consent form will include access to all clinical and laboratorial data. Genomic DNA will be extracted from blood samples using the QIAamp DNA Blood Midi Kit (Qiagen, Hilden, Germany) and DNA samples will be stored at −20°C. Quantification and quality analysis of DNA will be performed using NanoDrop 2000/c Spectrophotometer (ThermoFisher Scientific, Waltham, MA, USA). Polymerase chain reaction (PCR) will be performed with the oligonucleotides APOL1_7F (5′-CCAACTTTCTTTCCTTAGCTGGC-3′) and APOL1_7R (5′-TCACAGTTCTTGGTCCGCC-3′) as well as a PCR Master Mix kit (Promega, Madison, WI, EUA), in cycle conditions as described: 95°C for 5 min; 95°C for 30 s, 60°C for 30 s, and 72°C for 1 min (35 cycles); and 72°C for 10 min. Following PCR, 5 μL of each product will be subjected to electrophoresis on a 20% agarose gel and the amplicons will be identified based on the expected molecular weight. Th PCR product will be purified with Agencourt AMPure XP kit (Beckman Coulter Life Sciences, Indianapolis, IN, USA). Sanger sequencing will be performed with BigDye Terminator Cycle Sequencing Ready Reaction Kit version 3.1 (Applied Biosystems, Foster City, CA, USA) and an ABI Prism 3130XL automated sequencer (ThermoFisher Scientific, Waltham, MA, USA). The sequences will be compared with reference sequences (NG_023228) and confirmed by reverse strand sequencing. Data analysis will be performed with Software Chromas 2.6.6 (http://www.technelysium.com.au).

## Discussion

Renal involvement is common in cases of COVID-19. Previous studies have reported that up to 29% of infected patients may develop AKI and up to 59% of patients may develop proteinuria, which increases the risk of death (2, 21). Su et al. (14) propose that the disease induces early development of defective proteinuria and damages kidney tubular function. The mechanism by which this occurs remains unclear. Sepsis, acute tubular necrosis, cytokine storms, direct viral action, thrombosis, and rhabdomyolysis may be possible causes. ACE2 provides the entry point for SARS-CoV-2 to attach to and infect human cells; ACE2 is expressed on podocytes, vascular endothelial cells and proximal tubule cells. The ability of these different kidney cells to be infected may explain injuries develop in various parts of the kidneys (2–14).

In the literature, the presence of SARS-CoV-2 has been demonstrated using different techniques. Diao et al. (16) described the presence of syncytia as a finding associated with COVID-19 infection. Other authors, however, have not confirmed this finding (14). Moreover, viral particles can be difficult to unequivocally identify with EM. Additionally, the appearance of intracellular viral inclusions appears vary quite from one publication to another and must be differentiated from other structures present in the kidney tissue, mainly due to autolytic effects (22). In our study, all autopsies will be performed with a postmortem interval of <6 h, which might be short enough to prevent significant autolysis. Antigen detection by IHC is a very limited technique; this can be partly overcome with the aid, of other assays such as *in situ* hybridization. Detection of virus within renal cells by electron microscopy has been proposed as a straightforward approach to demonstrate direct renal involvement. However, for most authors, the identification of direct infection by SARS-CoV-2 does not exclude other mechanisms of renal injury in COVID-19 patients. In a case series reported by Kudose et al., electron microscopy, immunostaining for viral spike and nucleocapsid proteins, and ISH for viral RNA using an automated platform failed to demonstrate the presence of viral particles in renal tissue. The results of manual ISH were doubtful. However, the authors did not perform PCR, which is considered to have higher sensitivity for detecting viral material (17). Therefore, it is vital to use multiple techniques for reliable viral detection and to establish the role of direct viral action in different morphological patterns. Larger studies, such as the one we are currently proposing, on kidney biopsies of patients with COVID-19 are needed to definitively confirmed or rule out the existence of a possible SARS-CoV-2 nephropathy.

After the spread of the epidemic in the United States, reports of an association between collapsing glomerulopathy (a kind of podocytopathy), and SARS-CoV-2 infection were made. In some cases, the high-risk allele of *APOL1* was found; as previously described, this allele is present mainly in populations of African ancestry (7, 10). The large percentage of Afro-descendants present in the American population may explain why these types of cases did not occur in Asian and some European countries. It appears likely that in Afro-descended individuals with high-risk *APOL1* variants, SARS-CoV-2 infection acts as a “trigger” that leads to podocyte dysregulation and injury, which leads to CG. For some authors, however, this association may be just a coincidence (13) and the presence of the virus in podocytes may be an incidental finding. Moreover, a recent report of postmortem kidney tissue analysis from 6 patients who died of COVID-19 showed acute tubular injury without glomerular abnormalities (14).

Brazil is a country with a large population of Afro-descendants (around 40% of all Brazilians) (18) and the *APOL1* high-risk genotype found in Brazil was considered responsible for a more aggressive disease phenotype (23). In addition, the considerable genetic heterogeneity of the Brazilian population and the wide range of possible microenvironments may be highly advantageous for the study of genetic and environmental aspects associated with *APOL1*. These factors, along with Brazil's current critical situation in the face of the COVID-19 pandemic, make it the ideal country for conducting studies that assess the likely existence of a SARS-CoV-2-nephropathy.

AKI induced by SARS-CoV-2 also affects patients with previous renal disorders. Studies claim that chronic kidney disease (CKD) is associated with severe disease in those affected by COVID-19 (24). However, there are no large studies analyzing the impact of SARS-CoV-2 infection on patients with glomerulopathies; there are only case reports (25). Kidney transplant recipients appear to be a particularly high-risk group for critical COVID-19 illness due to chronic immunosuppression and coexisting conditions (25). Therefore, the impact of COVID-19 infection on transplant patients and patients followed up with on an outpatient basis will also be analyzed in our study.

In initial reports, renal injury was always associated with a severe pulmonary component. However, cases were later reported where renal injury did not depend on pulmonary involvement (24). In the present study, fragments of lung and liver will also be collected and the results will be compared with kidney specimens. With these results we hope to better understand the role of the coronavirus in the appearance and evolution of kidney injuries.

**Figure 4:**
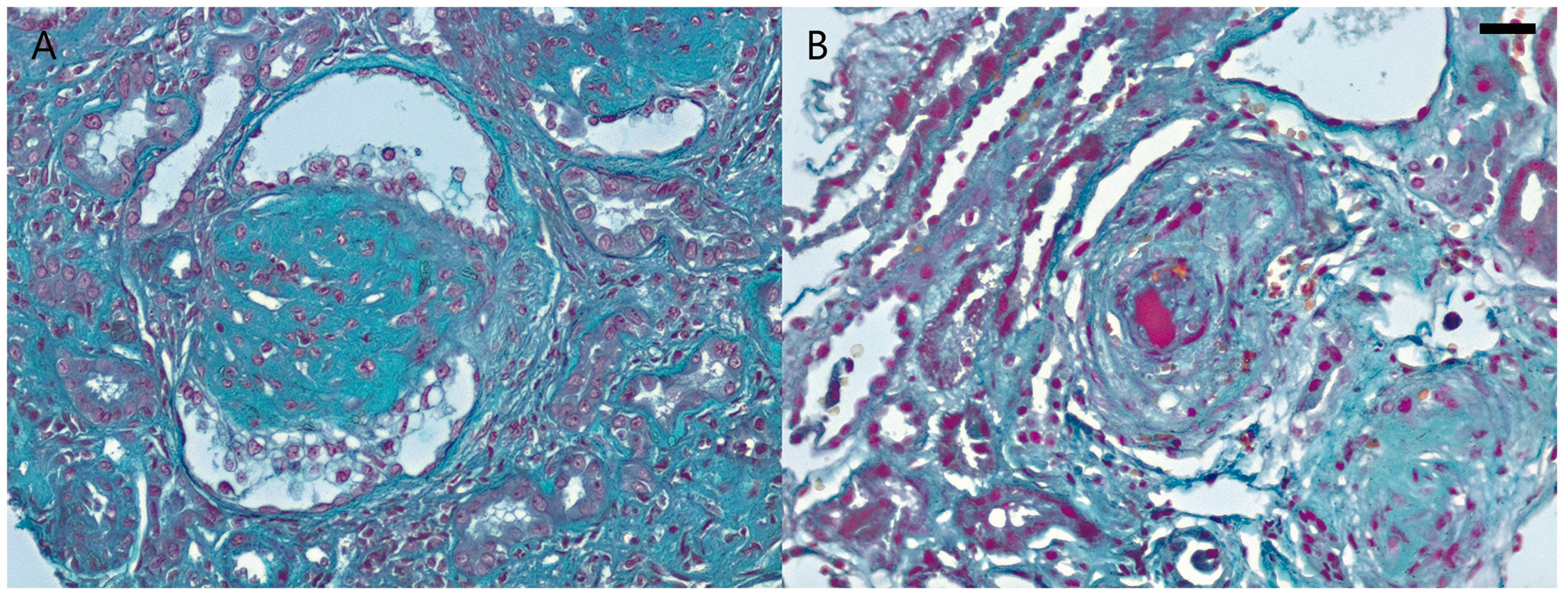
Renal morphologic patterns in COVID-19 patients from Empresa Brasileira de Serviços Hospitalares (EBSERH) network. **(A)** Needle biopsy in a male patient (aged 43 years) shows glomerulus with tuft collapse and overlying epithelial hypertrophy and hyperplasia compatible with collapsing glomerulopathy (Masson Trichrome stain; original magnification ×400). **(B)** Needle biopsy in a young male patient (aged 30 years) shows arterioles with intimal thickening and large luminal thrombus compatible with thrombotic microangiopathy (Masson Trichrome stain; original magnification ×400). Barr = 20 μm.

Here we included two morphological pictures described as SARS-Cov-2 nephropathy: a CG and a TMA (Figure 4). The two patients come from different hospitals from the *Empresa Brasileira de Serviços Hospitalares* (EBSERH) network. Both had a clinical picture compatible with COVID-19 and PCR was positive for SARS-CoV-2. The renal symptoms were noted after presentation of the symptoms of COVID-19 and both patients showed pulmonary involvement.

## Conclusion

Kidney injuries caused by SARS-CoV-2 are frequent and impact on morbidity and mortality, but considerable doubts remain about the likely injuries and their mechanisms. Ultimately, this work from the morphological point of view can help to define the spectrum and impact, whether immediate or long-term, of kidney injury caused by SARS-CoV-2.

## Ethics Statement

The studies involving human participants were reviewed and approved by Ethics Committee of the University Hospital of the Federal University of Maranhão. Written informed consent to participate in this study was provided by the participants' legal guardian/next of kin.

## Author Contributions

GS and NS: conceptualization. GS and AT: writing—review and editing. PN, JL, KC, MM, DB, AW, EW, LO, LLAN, AC, FB, GG, AM, DP, DM, TL, FG, OG, LV, KI, FL, LF, IM, GU, CM, LGLN, DW, SA, and WL: data or sample collection or histopathological analysis. All authors critically reviewed the manuscript.

## Conflict of Interest

The authors declare that the research was conducted in the absence of any commercial or financial relationships that could be construed as a potential conflict of interest.
